# Blast exposure elicits blood-brain barrier disruption and repair mediated by tight junction integrity and nitric oxide dependent processes

**DOI:** 10.1038/s41598-018-29341-6

**Published:** 2018-07-27

**Authors:** Aric F. Logsdon, James S. Meabon, Marcella M. Cline, Kristin M. Bullock, Murray A. Raskind, Elaine R. Peskind, William A. Banks, David G. Cook

**Affiliations:** 1 Geriatric Research Education and Clinical Center (GRECC), Veterans Affairs Puget Sound Health Care System, Seattle, WA 98108 USA; 2 Division of Gerontology and Geriatric Medicine, Department of Medicine, University of Washington School of Medicine, Seattle, WA 98195 USA; 3 Veterans Affairs Northwest Network, Mental Illness Research Education and Clinical Center, Veterans Affairs Puget Sound Health Care System, Seattle, WA 98108 USA; 4 Department of Psychiatry and Behavioral Science, University of Washington School of Medicine, Seattle, WA 98195 USA; 5 Department of Molecular and Cellular Biology, University of Washington, Seattle, WA 98195 USA

## Abstract

Mild blast-induced traumatic brain injury (TBI) is associated with blood-brain barrier (BBB) disruption. However, the mechanisms whereby blast disrupts BBB integrity are not well understood. To address this issue BBB permeability to peripherally injected ^14^C-sucrose and ^99m^Tc-albumin was quantified in ten brain regions at time points ranging from 0.25 to 72 hours. In mice, repetitive (2X) blast provoked BBB permeability to ^14^C-sucrose that persisted in specific brain regions from 0.25 to 72 hours. However, ^99m^Tc-albumin revealed biphasic BBB disruption (open-closed-open) over the same interval, which was most pronounced in frontal cortex and hippocampus. This indicates that blast initiates interacting BBB disruption and reparative processes in specific brain regions. Further investigation of delayed (72 hour) BBB disruption revealed that claudin-5 (CLD5) expression was disrupted specifically in the hippocampus, but not in dorsal striatum, a brain region that showed no blast-induced BBB permeability to sucrose or albumin. In addition, we found that delayed BBB permeability and disrupted CLD5 expression were blocked by the nitric oxide synthase inhibitor N(G)-nitro-L-arginine methyl ester (L-NAME). These data argue that latent nitric oxide-dependent signaling pathways initiate processes that result in delayed BBB disruption, which are manifested in a brain-region specific manner.

## Introduction

Mild traumatic brain injury (mTBI) associated with blast exposure is the most prevalent form of injury among military personnel who have served in Operation Enduring Freedom/Operation Iraqi Freedom/Operation New Dawn (OEF/OF/OND), with at least 300,000 personnel being subject to at least one exposure^1–5^. Mild TBI, termed an “invisible injury,” may increase the risk for neurodegenerative disorders in mid to late life^6–10^. Recently, the role of vascular disruption in neurodegenerative disorders and TBI has received more attention^11–13^.

Vascular injury, including disruption of the blood-brain barrier (BBB), may be an important initiating mechanism by which mTBI could trigger long-term pathophysiological cascades promoting neurodegeneration, including Alzheimer’s-like disorders and other neurodegenerative proteinopathies that include chronic traumatic encephalopathy^14–19^. BBB disruption has been examined in a number of pre-clinical models of blast-induced traumatic brain injury (TBI)^20–27^. However, the temporal dynamics and the anatomical vulnerability to blast-induced BBB disruption are still not well-understood. BBB opening has been shown to be a transient event following blast injury^23,28^, suggesting a physiological repair process. In addition, secondary processes that influence BBB integrity can develop that may not be due to damage from the simple mechanical forces of blast^23,29^.

In this study, we used a sensitive and highly quantifiable blood-borne radiolabeled tracer approach to assess the time course and magnitude of BBB disruption in multiple brain regions after blast-induced mTBI in mice. We chose time points to assess apparent mechanical damage (0.25 h), repair (3 and 24 h), and a hypothesized secondary process (72 h). We also assessed the effects of blast on tight junction integrity and the roles of nitric oxide and prostaglandin in mediating blast-induced BBB disruption.

## Results

### Blast exposure induces aberrant BBB permeability to radiolabeled sucrose and albumin

As an initial examination of the effects of blast exposure on BBB integrity, using well established methods^25^ we quantified specific BBB permeability to peripherally co-administered ^14^C-sucrose and ^99m^Tc-albumin that  under normal conditions cross the BBB poorly^30–32^. These molecules are often used to measure BBB permeability to small (~0.34 kDa) and larger (~66.44 kDa) blood-borne molecules, as well as for assessing aspects of paracellular and transcellular BBB disruption, respectively^33,34^.

**Figure 1:**
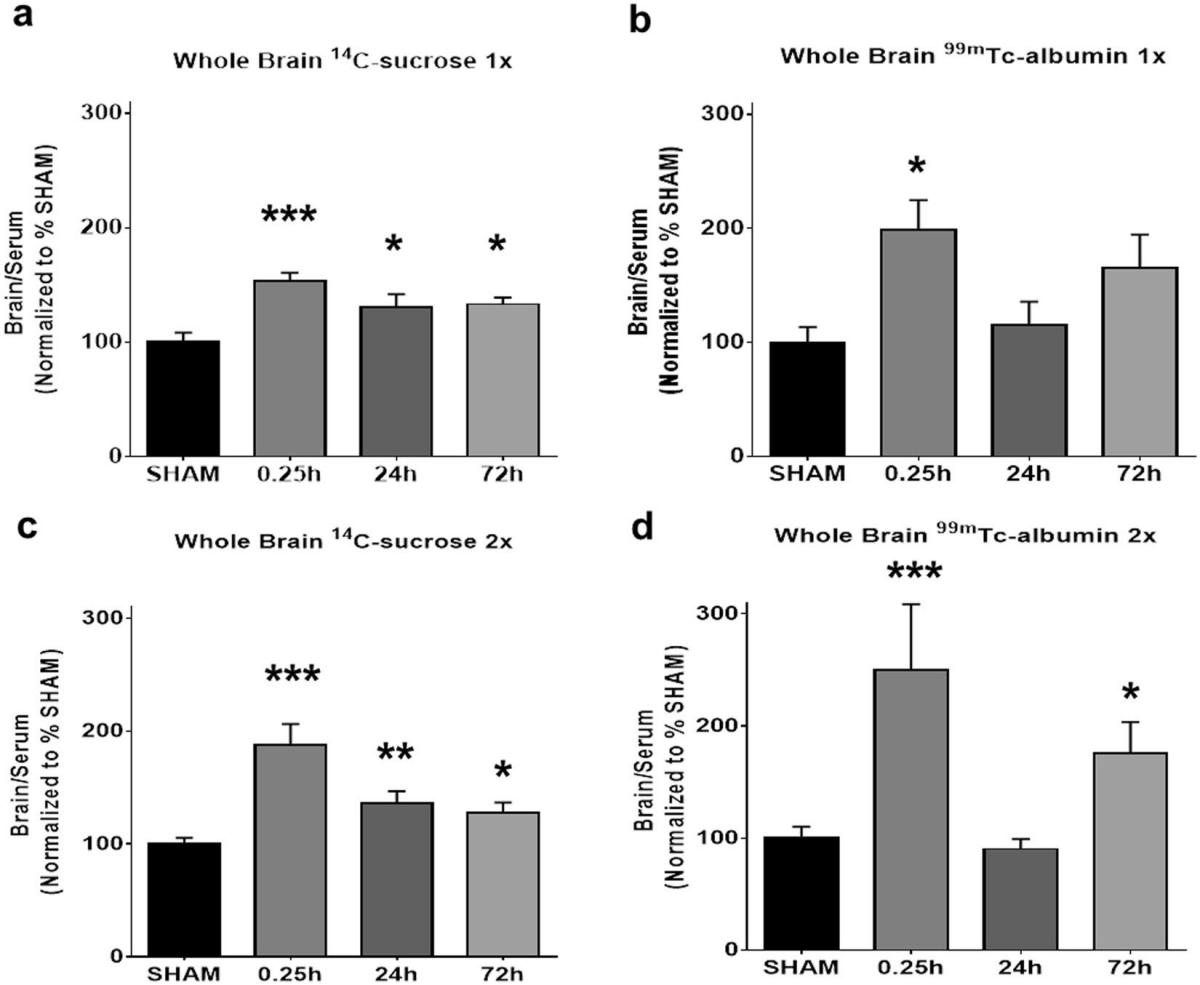
Blood-brain barrier disruption to large and small molecules following blast. **(a)** A significant increase in brain/serum ratios of ^14^C-sucrose, **(b)** and ^99m^Tc-albumin was observed in whole brain after 1X blast. **(c)** A significant increase in ^14^C-sucrose was also observed after 2X blast. **(d)** However, a significant increase in BBB permeability to albumin was at 0.25 h and 72 h after 2X blast, but not 24 h. One-way ANOVA *post-hoc* Dunnett’s. Values represent mean ± SEM. 1X blast: Sham (*n* = 7), mTBI 0.25 h (*n* = 8), 24 h (*n* = 8), and 72 h (*n* = 9). 2X blast: Sham (*n* = 22), mTBI 0.25 h (*n* = 5), 24 h (*n* = 11), and 72 h (*n* = 13). (*p ≤ 0.05; **p ≤ 0.01; ***p ≤ 0.001).

In keeping with previous reports^23,24,35^, blast overpressures were produced using a well-established pneumatic shock tube approach that accurately replicates battlefield-relevant shock waves generated by detonation of high explosives in the open field (Supplementary Fig. S1). A single blast exposure provoked BBB disruption as measured by ^14^C-sucrose entry into the CNS at 0.25, 24, and 72 h post-blast (Fig. 1a; *p* ≤ 0.01). Pair-wise Dunnett’s *post hoc* analysis confirmed a significant increase in ^14^C-sucrose CNS entry at 0.25 h (*p* ≤ 0.001, *n* = 8), 24 h (*p* ≤ 0.05; *n* = 8), and 72 h (*p* ≤ 0.05; *n* = 9) post-blast compared to sham controls (*n* = 7). The same mice also exhibited BBB permeability to ^99m^Tc-albumin (Fig. 1b; *p* ≤ 0.05). However, *post hoc* analysis revealed that blast-induced ^99m^Tc-albumin CNS entry was significantly elevated only at the shortest, 0.25 h time point (*p* ≤ 0.05) compared to sham controls. These results are in keeping with a level of BBB disruption due to a mild to moderate blast exposure that elicits both BBB injury and neuro-reparative processes. For the smaller sucrose molecules, BBB disruption persisted for at least 72 h, while aberrant BBB permeability to the larger albumin molecules was restored to normal levels within 24 h.

To assess the effects of repetitive injury, animals received two blasts (5 min inter-blast interval) that were of equivalent intensity to the 1X blast-exposed mice. These exposures occurred during a single, brief isoflurane treatment in order to minimize the number of anesthesia sessions needed for repetitive exposure (See Methods). 2X blast exposure evoked BBB permeability to ^14^C-sucrose that persisted for at least 72 h (Fig. 1c; *p* ≤ 0.001). *Post hoc* analysis confirmed a significant increase in ^14^C-sucrose at 0.25 h (*p* ≤ 0.001; *n* = 5), 24 h (*p* ≤ 0.01; *n* = 11) and 72 h (*p* ≤ 0.05; *n* = 13) post-blast compared to sham controls (*n* = 22). 2X blast also induced BBB permeability to ^99m^Tc-albumin (Fig. 1d; *p* ≤ 0.001). *Post hoc* analysis confirmed a significant increase in ^99m^Tc-albumin permeability at 0.25 h (*p* ≤ 0.001), as well as at 72 h post-blast (*p* ≤ 0.05) compared to sham controls.

The 2X blast results demonstrated that the CNS is able to restore normal BBB permeability to the larger albumin molecules within 24 h, while blast-induced BBB disruption affecting the sucrose radiotracer persisted for at least 72 h. However, unlike single blast, repetitive blast exposure initiated an additional latent process resulting in a delayed, second round of BBB permeability to ^99m^Tc-albumin that emerged by 72 h post-blast (Fig. 1d). Currently, little is understood regarding the mechanisms by which mild blast-induced neurotrauma gives rise to delayed BBB disruption. Therefore, we focused on the 72 h opening to ^99m^Tc-albumin occurring after 2X blast.

### Blast elicits brain region-specific BBB disruption

To investigate the vulnerability of distinct brain regions to repetitive blast-induced BBB disruption, we quantified aberrant ^14^C-sucrose and ^99m^Tc-albumin BBB permeability in ten brain regions: frontal cortex, occipital cortex, parietal cortex, striatum, midbrain, thalamus, cerebellum, pons/medulla (brainstem), and hippocampus. Eight of the ten anatomical regions displayed significant blast-induced permeability to blood-borne ^14^C-sucrose within 0.25 h compared to sham controls (Table 1). Under these conditions, blast exposure provoked significant BBB disruption that persisted in four of ten brain regions at 3 h (*p* ≤ 0.05), three of ten regions at 24 h (*p* ≤ 0.05), and four of ten regions at 72 h (*p* ≤ 0.05). Of note, the hippocampus and frontal cortex exhibited biphasic permeability to ^14^C-sucorse at 0.25 and 72 h, but not at 24 h. 2X blast exposure significantly increased BBB permeability to ^99m^Tc-albumin in the hippocampus, frontal cortex, and pons/medulla compared to sham controls (Table 2). For these brain regions, normal BBB integrity against ^99m^Tc-albumin entry was restored within 3 to 24 h; and was followed by a second cycle of BBB disruption at 72 h. Similar to the sucrose data (Table 1), the hippocampus and frontal cortex exhibited biphasic permeability to albumin at 0.25 and 72 h. Of special note was an increase in BBB opening at 72 h that occurred in the pons-medulla (and whole brain) for ^99m^Tc-albumin but not for ^14^C-sucrose (Table 2). Thus, blast exposure elicited a multiphase CNS response involving anatomically distinct manifestations of BBB disruption, initiating both immediate and latent processes that differentially modulated permeability to smaller and larger blood-borne molecules.

**Table 1:** Temporal profile of regional BBB permeability to ^14^C-sucrose after 2X blast mTBI.

Brain region	0.25 h (*n* = 5)	3 h (*n* = 12)	24 h (*n* = 11)	72 h (*n* = 13)
Frontal cortex	104 ± 17^***^	33 ± 12^*^	30 ± 13	33 ± 12^*^
Occipital cortex	70 ± 16^***^	40 ± 11^**^	31 ± 12^*^	22 ± 11
Parietal cortex	90 ± 17^***^	43 ± 12^**^	40 ± 13^*^	29 ± 12
Striatum	30 ± 23	20 ± 17	33 ± 17	13 ± 16
Midbrain	82 ± 16^***^	27 ± 12	27 ± 12	23 ± 11
Thalamus	17 ± 16	10 ± 12	27 ± 12	34 ± 11^*^
Cerebellum	48 ± 15^*^	21 ± 11	26 ± 12	27 ± 11
Pons-medulla	139 ± 21^***^	54 ± 15^**^	44 ± 15^*^	38 ± 15^*^
Hippocampus	53 ± 15^**^	06 ± 11	18 ± 11	28 ± 10^*^
Hypothalamus	66 ± 21^*^	12 ± 15	15 ± 16	23 ± 15
Whole Brain	87 ± 15^***^	34 ± 11^*^	36 ± 11^*^	28 ± 11^*^

Blood-brain barrier (BBB); mild traumatic brain injury (mTBI). Values represent mean difference ± SEM normalized to sham control animals (*n* = 22). One-way ANOVA; *post-hoc* Dunnett’s; *p ≤ 0.05; **p ≤ 0.01; ***p ≤ 0.001.

**Table 2:** Temporal profile of regional BBB permeability to ^99m^Tc-albumin after 2X blast mTBI.

Brain region	0.25 h (*n* = 5)	3 h (*n* = 12)	24 h (*n* = 11)	72 h (*n* = 13)
Frontal cortex	289 ± 77^***^	59 ± 56	−18 ± 57	148 ± 54^*^
Occipital cortex	69 ± 56^***^	73 ± 41	−21 ± 42	10 ± 39
Parietal cortex	129 ± 58^***^	62 ± 42	−09 ± 43	65 ± 41
Striatum	29 ± 61	−19 ± 44	−24 ± 47	−12 ± 43
Midbrain	69 ± 56^***^	73 ± 41	−21 ± 42	10 ± 40
Thalamus	16 ± 79	107 ± 57	17 ± 59	153 ± 55^*^
Cerebellum	48 ± 35^*^	16 ± 25	−20 ± 26	29 ± 24
Pons-medulla	120 ± 41^***^	57 ± 30	11 ± 30	88 ± 29^*^
Hippocampus	203 ± 72^**^	−31 ± 52	−27 ± 54	144 ± 51^*^
Hypothalamus	66 ± 73^*^	18 ± 53	−18 ± 55	59 ± 52
Whole Brain	150 ± 36^***^	30 ± 26	−10 ± 27	75 ± 26^*^

Blood-brain barrier (BBB); mild traumatic brain injury (mTBI). Values represent mean difference ± SEM normalized to sham control animals (*n* = 22). One-way ANOVA; *post-hoc* Dunnett’s; *p ≤ 0.05; **p ≤ 0.01; ***p ≤ 0.001.

### Delayed BBB disruption is associated with brain region-specific disturbances in claudin-5 expression

One mechanism by which delayed BBB disruption could have occurred is through altered expression of the brain endothelial cell (BEC) tight junction protein, claudin-5 (CLD5). Our group has previously shown aberrant CLD5 expression in the acute phase following blast mTBI^23^. If disturbed CLD5 expression plays a role in mediating delayed blast-induced BBB disruption, we expected to find both a time and brain region-specific disturbance in the pattern of CLD5 expression mirroring the functional BBB integrity pattern seen above.

**Figure 2:**
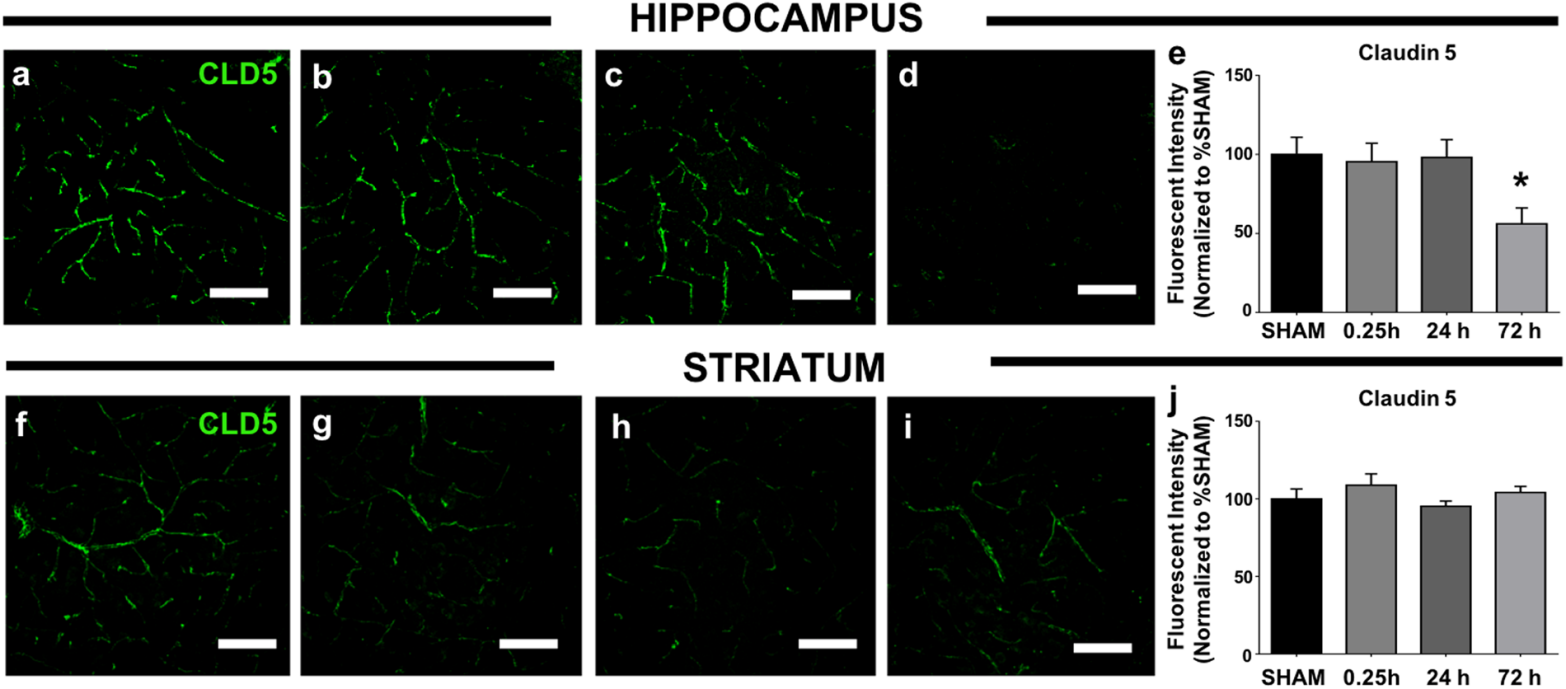
Claudin-5 expression is disturbed in brain regions vulnerable to blast-induced BBB disruption. **(a)** Shows representative images of CLD5 immunofluorescence (green) in sham hippocampal CA1, **(b)** at 0.25 h, **(c)** 24 h, and **(d)** 72 h after 2X blast. **(e)** Fluorimetric quantitation conducted by a condition-blind rater revealed a significant decrease in CLD5 fluorescence measured at 72 h after 2X blast. **(f)** Shows representative images of sham CLD5 immunofluorescence in dorsal striatum, **(g)** at 0.25 h, **(h)** 24 h, and **(i)** 72 h after 2X blast. **(j)** Fluorimetric quantitation revealed no significant differences in CLD5 fluorescence after 2X blast. One-way ANOVA *post hoc* Dunnett’s for CLD5. Values represent mean ± SEM; *n* = 4. (*p ≤ 0.05). Scale bars = 50 µm.

The hippocampus displayed biphasic permeability to both ^14^C-sucrose and ^99m^Tc-albumin following blast exposure (Tables 1 and 2). Conversely, the striatum displayed the least vulnerability to blast-induced BBB disruption (Tables 1 and 2). Therefore, we focused our attention on these regions. CLD5 immunoreactivity in hippocampal CA1 appeared markedly reduced 72 h post-blast (*n* = 4/4) compared to sham controls (Fig. 2a–d); *n* = 0/4). The intensity of CA1 CLD5 immunoreactivity was quantified using Imaris software (see Methods) by an operator who was blind to the experimental conditions. 2X blast provoked a 43.9% loss of CLD5 immunoreactivity in the CA1 region of hippocampus at 72 h compared to sham controls (Fig. 2e; *p* ≤ 0.05). In contrast to the hippocampus, CLD5 immunoreactivity in the dorsal striatum of these same mice used was not significantly altered by 2X blast exposure at any time point (Fig. 2f–j).

The loss of CLD5 immunoreactivity in hippocampus at 72 h could be due either to a loss of overall CLD5 protein levels or to mislocalization/diffusion of CLD5 away from the prominent tight junction domains where CLD5 is highly concentrated. Western blot analysis of CLD5 levels revealed no significant changes in hippocampal, or striatal protein lysates of 2X blast mice versus sham controls (Supplementary Fig. S2). Thus, as has been reported previously regarding CLD5 expression after brain injury^36–38^, it is more likely blast caused extant CLD5 proteins to mislocalize and diffuse within the endothelial cytoplasm, making them more difficult to detect by immunofluorescence. Taken together these results indicate that blast exposure caused brain region-specific disturbances in the expression of tight junction-localized CLD5. Interestingly, we found no changes in CLD5 immunoreactivity at 0.25 h post-blast, even though there was a significant increase in blast-induced permeability to ^14^C-sucrose and ^99m^Tc-albumin (Fig. 1, Tables 1 and 2). This suggests that the mechanisms mediating BBB disruption immediately after blast are distinct from second phase BBB disruptions arising 72 h later.

### Blast causes perivascular reactive gliosis in association with aberrant claudin-5 expression

**Figure 3:**
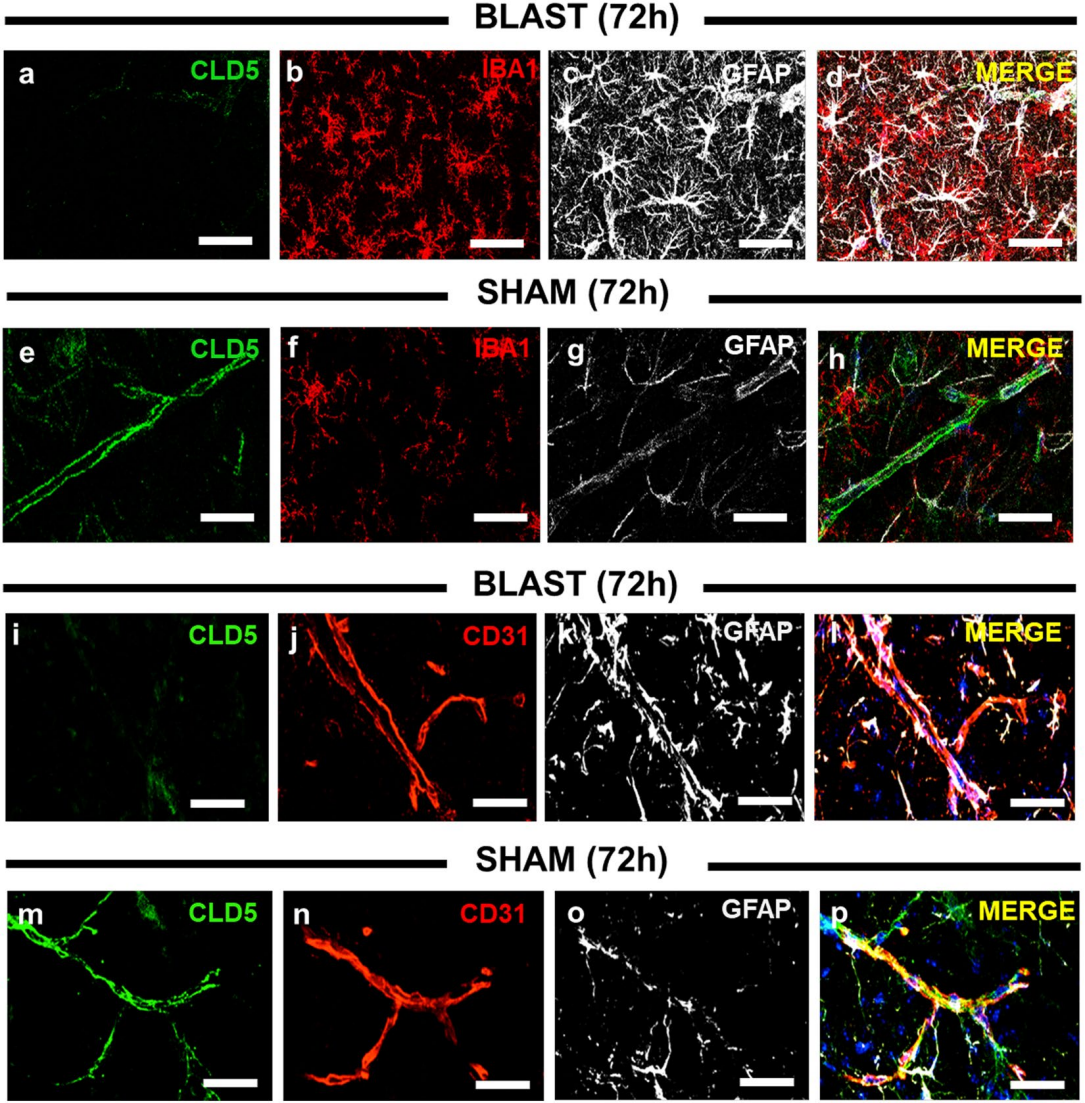
Perivascular reactive gliosis occurs in association with aberrant claudin-5 expression after blast. **(a)** BLAST panels (top) show representative reduced tight junction protein (CLD5) immunofluorescence, **(b)** with markedly increased microglia (IBA1), **(c)** and astrocyte (GFAP) immunofluorescence in the frontal cortex. **(d)** MERGE (red, green, and white) shows the collective overlay of the neurovascular unit in the frontal cortex at 72 h after 2X blast. **(e)** SHAM panels (top) show representative images of intact CLD5, **(f)** with low microglia, **(g)** and astrocyte reactivity. **(h)** Neurovascular unit in the frontal cortex of a sham control. **(i)** BLAST panels (bottom) show representative reduced CLD5 immunofluorescence, **(j)** intact blood vessels (CD31), **(k)** increased GFAP immunofluorescence, **(l)** and the neurovascular unit in the frontal cortex at 72 h after 2X blast. **(m)** SHAM panels (bottom) show representative images of intact CLD5, **(n)** intact blood vessels, **(o)** low GFAP immunofluorescence, **(p)** and the neurovascular unit in the frontal cortex of a sham control. Scale bars = 30 µm.

As with hippocampus, a delayed increase in permeability to both ^14^C-sucrose and ^99m^Tc-albumin at 72 h post-blast was also observed in frontal cortex (Tables 1 and 2). In keeping with this, we similarly observed decreased CLD5 immunoreactivity in frontal cortex of blast-exposed mice (Fig. 3a and i; *n* = 4/4) compared to sham controls (Fig. 3e and m; *n* = 0/4). Triple-label confocal immunostaining of ionized calcium-binding adapter molecule 1 (IBA1) and glial fibrillary acidic protein (GFAP) revealed increased reactive gliosis at 72 h post-blast (Fig. 3b–d; *n* = 4/4) compared to sham controls (Fig. 3f–h; *n* = 0/4). CD31 (an endothelial cell-specific marker) showed that BECs appeared intact (Fig. 3j and n); even though their CLD5 immunoreactivity was markedly reduced in blast-exposed mice. Additional GFAP immunostaining confirmed perivascular reactive gliosis at 72 h post-blast (Fig. 3k,l; *n* = 4/4) compared to sham controls (Fig. 3o,p; *n* = 0/4). In spite of robust morphological disturbances in the BECs and perivascular astrocytes, we found no overt alterations in neuronal somato-dendritc morphology at 72 hours post-blast (Supplementary Fig. S3). This is broadly in keeping with previous findings in this animal model in other brain regions where it takes a month, or more for significant neuronal loss to become evident^24^.

Taken together, these data confirm the potential significance of disturbed CLD5 expression during delayed BBB disruption and highlight the comparative vulnerability of the BBB to blast exposure. These data also show that aberrant CLD5 expression is closely associated with reactive gliosis, thus suggesting that perivascular neuroinflammation may play a role in delayed aberrant CLD5 expression provoked by blast exposure.

### Effects of cyclooxygenase or nitric oxide synthase inhibition on delayed BBB disruption

**Figure 4:**
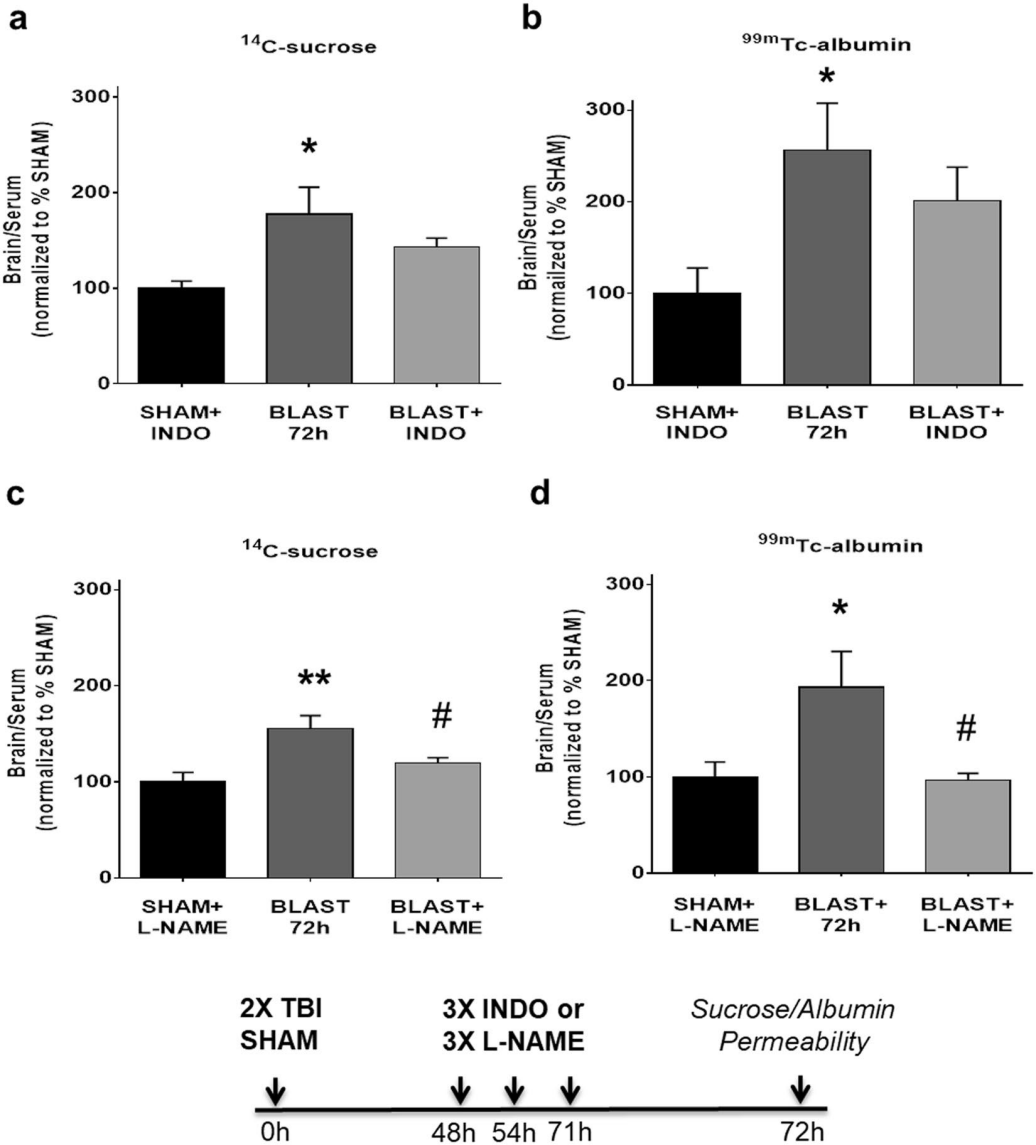
Nitric oxide synthase inhibition blocks delayed blood-brain barrier disruption after blast. **(a)** At 72 h after 2X blast, a significant increase in ^14^C-sucrose, **(b)** and ^99m^Tc-albumin was measured in whole brains compared to sham controls treated with INDO (5 mg/kg; ip). **(a)** No significant differences in ^14^C-sucrose, **(b)** or ^99m^Tc-albumin were observed when INDO was administered after blast. **(c)** A significant increase in ^14^C-sucrose, **(d)** and ^99m^Tc-albumin was also observed in whole brain at 72 h after 2X blast compared to L-NAME treated sham controls. **(c)** L-NAME administration (10 mg/kg; ip) significantly attenuated a blast-induced increase in ^14^C-sucrose, **(d)** and ^99m^Tc-albumin. One-way ANOVA *post hoc* Newman-Keul’s. Values represent mean ± SEM; *n* = 5. (*p ≤ 0.05 vs sham + L-NAME; ^#^*p* ≤ 0.05 vs blast + vehicle; **p ≤ 0.01 vs sham + INDO).

Broadly speaking, numerous findings point to at least two primary neuroinflammatory signaling cascades, the prostaglandin and/or nitric oxide pathways, which could mediate aspects of blast-induced delayed BBB disruption^39–41^. We first tested the potential significance of prostaglandin-related signaling pathways by inhibiting cyclooxygenase with indomethacin (INDO). To accomplish this, we administered INDO at 48, 54, and 71 h post-blast and then measured BBB permeability to ^14^C-sucrose and ^99m^Tc-albumin at 72 h. As expected, blast exposed mice (*n* = 5) exhibited a significant increase of 77.4% in ^14^C-sucrose permeability at 72 h (Fig. 4a; *p* ≤ 0.05) compared to INDO sham controls (*n* = 5). However, INDO treatment did not significantly reduce the effects of blast on BBB permeability to ^14^C-sucrose. Blast exposure also increased BBB permeability to ^99m^Tc-albumin (Fig. 4b), with a significant 156% increase in ^99m^Tc-albumin at 72 h post-blast (*p* ≤ 0.05) compared to INDO sham controls. As with ^14^C-sucrose, INDO treatment only partially reduced BBB permeability to ^99m^Tc-albumin. These results suggest that under these experimental conditions prostaglandin-related pathways play a limited role in mediating second phase BBB disruption caused by blast exposure.

We next treated mice with N(G)-nitro-L-arginine methyl ester (L-NAME), a well-characterized pan-nitric oxide synthase (NOS) inhibitor^42–46^. L-NAME was injected at 48, 54, and 71 h post-blast or sham treatment and brain BBB permeability was assessed at 72 h. Blast exposed mice (*n* = 5) exhibited a significant increase of 55% in ^14^C-sucrose permeability at 72 h (Fig. 4c; *p* ≤ 0.01) compared to L-NAME sham controls (*n* = 5). Moreover, L-NAME significantly reduced ^14^C-sucrose permeability by 35.8% compared to vehicle-treated/blast exposed animals (Fig. 4c; *p* ≤ 0.05). 2X blast also increased ^99m^Tc-albumin permeability at 72 h (Fig. 4d). A significant 93% increase in ^99m^Tc-albumin was measured in whole brain at 72 h post-blast (*p* ≤ 0.05) compared to L-NAME sham controls. L-NAME significantly reduced ^99m^Tc-albumin permeability by 96% compared to vehicle-treated/blast exposed animals (Fig. 4d; *p* ≤ 0.05). These data argue that the mechanisms governing delayed blast-induced BBB disruption involve one or more nitric oxide signaling pathways that are engaged over a period of 48 to 72 h following injury.

### L-NAME reverses blast-induced disturbances in claudin-5 expression

**Figure 5:**
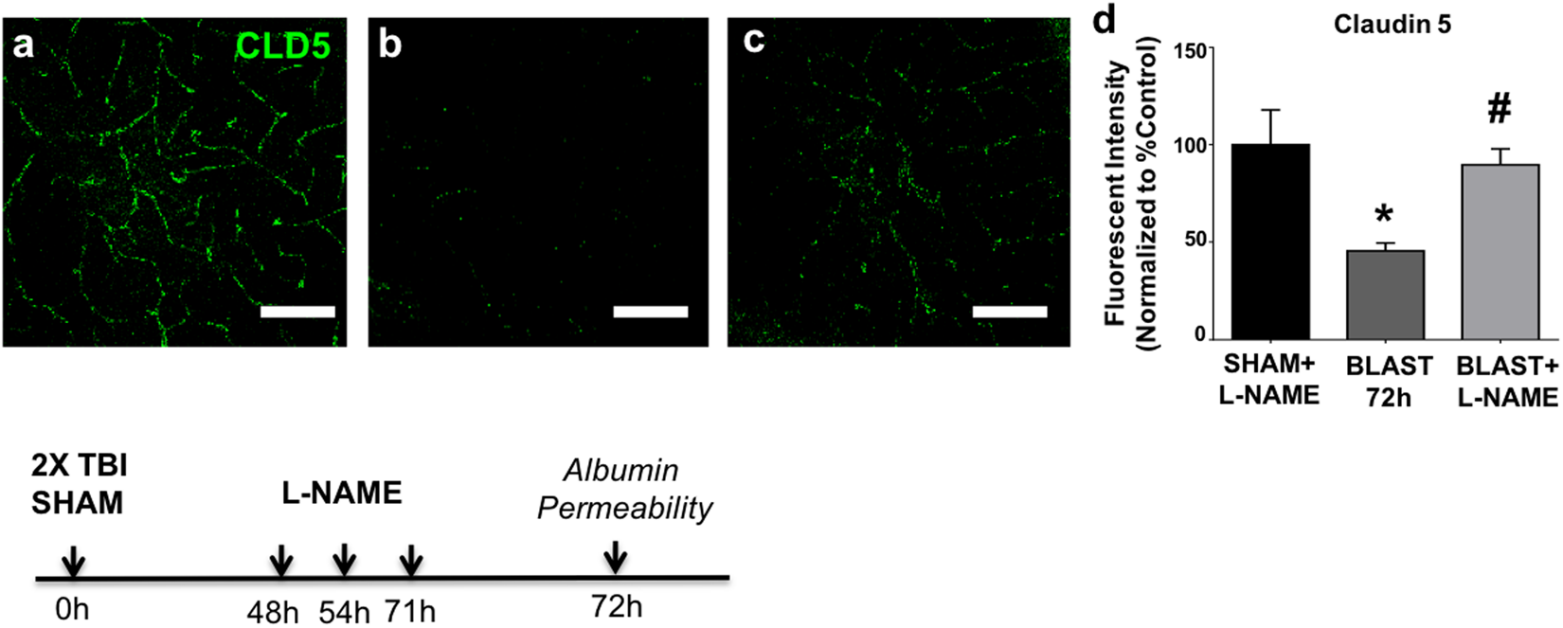
L-NAME rescues claudin-5 immunofluorescence in the hippocampus after blast. **(a)** Shows representative sham control CLD5 immunostaining in hippocampal CA1 + L-NAME (10 mg/kg; ip), **(b)** at 72 h after 2X blast + vehicle, and **(c)** at 72 h after 2X blast + L-NAME. **(d)** A significant decrease in CLD5 immunofluorescence was measured at 72 h after 2X blast + vehicle, which was rescued by L-NAME administration. One-way ANOVA *post hoc* Newman-Keul’s. Values represent mean ± SEM; *n* = 4. (*p ≤ 0.05 vs sham + vehicle, and sham + L-NAME; ^#^*p* ≤ 0.05 vs blast + vehicle). Scale bars = 50 µm.

The ability of L-NAME to rescue delayed blast-induced BBB disruption led us to hypothesize that L-NAME may block blast-induced disturbances in CLD5 expression. Representative CLD5 confocal microscopy results show that CA1 hippocampal CLD5 immunoreactivity was reduced in 72 h post-blast mice (*n* = 4/4) compared to a sham controls (Fig. 5a,b); *n* = 0/4). CLD5 immunostaining appeared normal in blast-exposed animals treated with L-NAME (Fig. 5c; *n* = 4/4). Quantification of CLD5 immunoreactivity by a condition-blind observer revealed a significant 54% decrease in CLD5 immunofluorescence intensity in 72 h post-blast mice compared to sham controls (Fig. 5d; *p* ≤ 0.05). L-NAME administration to blast mice significantly rescued CLD5 immunofluorescence by 44% compared to vehicle-treated/blast exposed animals (Fig. 5d; *p* ≤ 0.05).

We also examined hippocampal CA1 CLD5 immunostaining in blast exposed mice treated with INDO. In contrast to the effects of L-NAME, INDO treatment failed to normalize CLD5 expression in hippocampus (Supplementary Fig. S4). Overall, these data significantly strengthen the idea that nitric oxide-related signaling pathways mediate second-phase BBB disruption and CLD5 disturbances caused by blast.

### L-Name attenuates TNFα in BBB-vulnerable brain regions after blast

**Figure 6:**
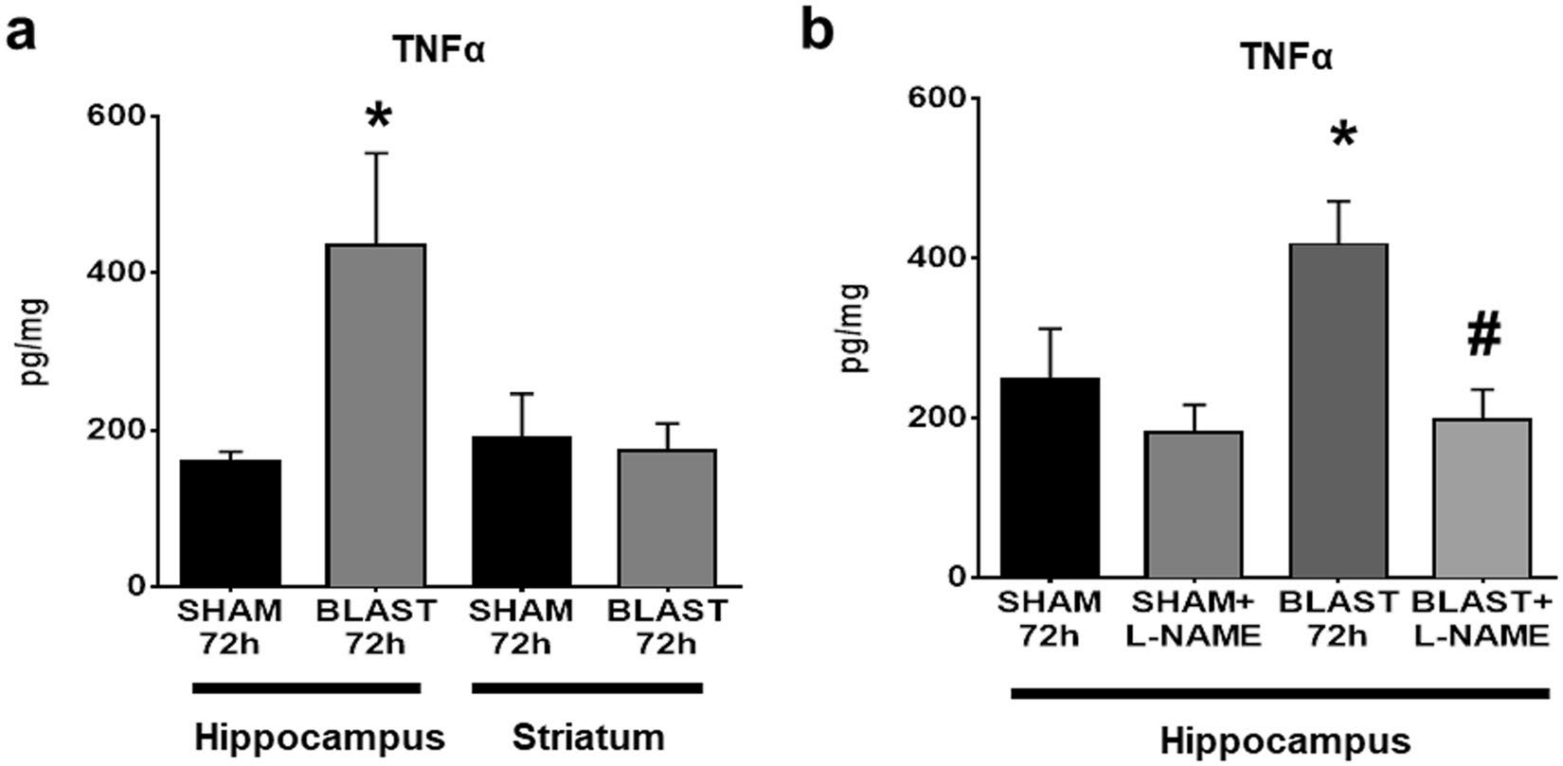
Brain region-specific blast-induced increases in TNFα are attenuated by L-NAME. **(a)** A significant increase in TNFα protein expression was measured hippocampus, but not in striatum at 72 h after 2X blast. **(b)** L-NAME (10 mg/kg; ip) attenuated a blast-induced increase in hippocampal TNFα. One-way ANOVA *post hoc* Newman-Keul’s. Values represent mean ± SEM; *n* = 5. (*p ≤ 0.05 vs sham + vehicle, and sham + L-NAME; ^#^*p* ≤ 0.05 vs blast + vehicle).

TNFα is known to alter CLD5 expression^47^. To test whether blast induces brain region-specific changes in TNFα, we measured TNFα in hippocampus and striatum at 72 h. Hippocampal brain lysates from blast exposed mice (*n* = 5) exhibited a significant increase of 276.2 ± 118.4 (mean ± SEM) in TNFα protein expression (pg/mg) compared to sham controls (Fig. 6a; *p* ≤ 0.05; *n* = 5). No significant differences were observed in the striatum of the same mice (Fig. 6a).

A complex interplay exists between nitric oxide and TNFα^48–51^; therefore, in a separate experiment we examined the effects of L-NAME treatment on blast-induced TNFα expression in the hippocampus. A significant difference in TNFα protein expression was observed at 72 h after 2X blast (Fig. 6b; *p* ≤ 0.05). Blast exposed mice (*n* = 5) exhibited an increase of 168.8 ± 82.9 in TNFα protein expression compared to sham controls (*n* = 5), and an increase of 235.3 ± 63.9 compared to L-NAME sham controls (*n* = 5). L-NAME administration after blast (*n* = 5) significantly decreased TNFα protein expression by 219.8 ± 65.7 compared to vehicle-treated/blast exposed animals (Fig. 6b; *p* ≤ 0.05), suggesting that the mechanism by which blast provokes delayed BBB disruption involves nitric oxide-mediated TNFα elevation. Moreover, these data also suggest that the mechanistic pathways regulated by NOS and TNFα may play a role in accounting for differences in the vulnerability of different brain regions (in this instance, the hippocampus versus the striatum) to delayed cycles of blast-induced BBB disruption.

## Discussion

In keeping with other reports^21,23,52^, we found that a single blast exposure provokes rapid, transient BBB disruption, permitting aberrant entry of both sucrose and albumin radiotracers into multiple brain regions. By 24 h, BBB integrity was restored to albumin, but not sucrose. After two consecutive (2X) mild blast exposures, the BBB reopens to albumin at 72 hours. We found that tight junction disintegrity correlates anatomically and temporally to this second phase of BBB disruption. These data report that blast exposure causes immediate and sustained brain permeability to small molecules, such as sucrose and is accompanied by additional latent processes that elicit delayed, brain region-specific permeability to larger molecules. Complementary findings of biphasic BBB opening has been reported in feline^53^ and murine models of stroke^54,55^. Furthermore, biphasic BBB opening has been observed in rats after controlled cortical impact TBI^56,57^.

Taken together, these findings suggest that several different forms of brain trauma set in motion multiple physiological and/or pathological processes with interacting phases of BBB repair and delayed disruption. Thus, as is well-established in managing the pathologic processes attending stroke, these results highlight the importance of elucidating the specific pathophysiological cascades evoked by blast, as well as the timing of these cascades. Both considerations are crucial in the search for appropriate therapeutic targets; and in establishing when such interventions would likely to be of benefit rather than potential harm.

Previously we have reported that inhibiting prostaglandin synthesis with indomethacin can reduce lipopolysaccharide-induced BBB permeability^39^. Other groups have shown that inhibition of nitric oxide production with L-NAME can reduce stroke-induced BBB permeability^58^. In this study, we show that secondary BBB disruption following blast is significantly attenuated by pan-specific NOS signaling inhibition with L-NAME. The results suggest that NOS pathways play an important role in mediating tight junction integrity after neurotrauma and have potential therapeutic implications for reducing BBB dysfunction and subsequent pathophysiology after blast injury. It has been shown that tight junction remodeling can occur after BEC perturbation^59–63^, which could explain why albumin CNS influx is attenuated by 24 hours. Initial BBB opening is likely dominated by mechanical disruption of endothelial membranes caused by a putative surge of vascular overpressure as previously suggested^64^ and is consistent with reported vascular pathology^12^. It may also be possible that the BBB continues to oscillate between open and closed into the subacute phase, and this may be due to ongoing aberrant nitric oxide production induced by blast.

It is possible that NO production could subserve BEC compensatory mechanisms that cause tight junction protein mislocalization, and upregulates caveolin-1-dependent transcytosis^46^. This could partially explain why L-NAME reduces albumin influx, suggesting that future studies to address the causal mechanism(s) by which blast opens the BBB to larger molecules. The nitric oxide synthase pathway is activated after blast TBI^52,65^ and has been implicated in neurodegeneration associated with TBI^66,67^. Moreover, nitric oxide inhibition has been shown to improve sensorimotor function^68^, reduce neuronal cell death^69^, and prevent edema formation after TBI^70^, supporting the importance of nitric oxide pathways in response to brain injury^71^.

Tight junction protein expression has been shown to change after blast injury^22,23^ and correlates with TBI-induced BBB disruption^64,72^. Our findings of aberrant CLD5 expression 72 hours after blast, without a significant change in CLD5 protein expression as measured by immunoblotting, could suggest that CLD5 may not be degraded as a result of blast, but rather diffused within BECs. Such redistribution has been demonstrated in rodent stroke models^36,37^ and in primary BECs after blast injury^38^. CLD5^−/−^ mice exhibited BBB disruption to small molecules, such as sucrose, with no effect on larger molecules such as albumin^73^. This raises the possibility that aberrant CLD5 expression alone may not fully account for the delayed phase of blast-induced albumin influx into the CNS.

Perivascular astrocytic end-feet surround the abluminal side of BECs and are necessary for the formation and maintenance of the tight junctional barrier and BBB integrity^74,75^. In an animal model of CNS inflammatory disease, endothelial growth factors released by activated astrocytes have been shown to reduce tight junction integrity, inducing BBB breakdown^76,77^. Thus, our findings of marked perivascular reactive gliosis and brain region-specific increases in TNFα argue that intercellular interactions between astrocytic neuroimmune responses and the BECs they enshroud, play important roles in mediating the anatomical and temporal manifestations of blast-induced BBB disruption.

Veterans with mild TBI show structural and functional CNS abnormalities^78–82^. Future studies will focus on vulnerable brain regions at prolonged time points to assess the pathophysiology accompanying BBB disruption. Vulnerable regions of the BBB may be those that are more susceptible to chronic low-grade inflammation and associated with long-term neurodegeneration. The results herein build upon very recent findings of regional blast-induced BBB disruption^83^ also suggest that inhibiting nitric oxide may block the cyclic nature of BBB disruption such that carefully targeted and timed therapies may help prevent long-term blast-related pathophysiology, cognitive, and behavioral disturbances.

## Methods

### Animals

Male C57BL/6 (*n* = 189) mice (Jackson Laboratories) aged 8–12 weeks (27–32 g) were used for this study. All mice had *ad libitum* access to food and water, were maintained under a 12 h day/night cycle, and group housed. Animals were randomly assigned to sham, drug, or mTBI groups. All animals were housed and handled in accordance with protocols approved by the Veterans Affairs Puget Sound Health Care System’s Institutional Animal Care and Use Committee (IACUC) and all experiments were conducted in accordance with the National Institutes of Health Guide for the Care and Use of Laboratory Animals. All experiments reported are in compliance with the ARRIVE guidelines.

### Blast exposure

All mice were allowed to acclimatize to the animal facility for at least one week prior to blast exposure. In preparation for blast exposure, animals were first anesthetized with 5% isoflurane, followed by maintenance with 2% isoflurane using a non-rebreathing anesthesia machine at a flow rate of 1 L/min oxygen. A flexible custom facemask designed to fit over the nose and mouth was attached to the mouse harness and provided anesthesia during blast exposure or sham treatment. In keeping with well-established procedures^23,35^, mice were positioned in the shock tube with their ventral body surface facing the oncoming shock wave as previously described. A pneumatic shock tube was used to expose mice to either a single blast exposure over the course of 6–7 min, or two consecutive blast exposures over the course of 12–14 min. Two consecutive blast exposures consistently produced a significant effect at 72 h (*p* ≤ 0.05), compared to a more variable response after a single blast. After the first blast exposure, the mouse was removed from the shock tube and re-adjusted in the harness prior to the second exposure. Sham control animals were mounted in the restraint harness and held under anesthesia for the same amount of time as the respective blast-exposed mice.

The blast exposure used in these experiments had a peak static pressure of 20.56 psi (±0.21), a positive phase duration of 5.37 ms (±0.070), and a shock wave velocity of 1.4 Mach. In keeping with a mild to moderate blast exposure 176/189 animals in this study survived the blast exposure (1X or 2X) and by inspection displayed normal ambulation, visually guided grasping responses when elevated by the tail near a foothold, and grooming behavior within 1 hour after exposure.

### Radiolabeled tracer preparation

Following established procedures^39^, albumin (Sigma, St. Louis MO) was labeled with ^99m^Tc (GE Healthcare, Piscataway, NJ). A mixture of 240 mg/ml stannous tartrate and 1 mg/ml albumin was adjusted to pH 3.0 with HCl. One millicurie of ^99m^Tc-NaOH_4_ was added to this mixture and allowed to incubate for 20 min. The ^99m^Tc-albumin was purified on a column of G-10 Sephadex (GE Healthcare) in 0.1 ml fractions of phosphate buffer (0.25 M). Radioactivity in the purified ^99m^Tc-albumin peak was more than 90% acid precipitable in an equal volume of 1% bovine serum albumin (BSA) and trichloroacetic acid (30%). 5 × 10^6^ cpm/mouse of purified ^99m^Tc-albumin fraction was combined with 1 × 10^7^ dpm/mouse of ^14^C-sucrose (Perkin Elmer, Waltman, MA) in a final volume (0.2 ml/mouse) of lactated Ringer’s solution containing 1% BSA.

### Radiolabeled tracer injections

At 0.25, 24, and 72 h after single blast, and at 0.25, 3, 24, and 72 h after 2X blast, or after time-matched sham procedures, mice were anesthetized with urethane (4 g/kg; 0.2 ml; ip), and the jugular veins exposed. The mice were given an intravenous co-injection of ^99m^Tc-albumin (5 × 10^6^ cpm) + ^14^C-sucrose (1 × 10^7^ dpm) in 0.2 ml of lactated Ringer’s solution with 1% BSA for 10 min. Blood was collected from a cut in the descending abdominal aorta. The vascular space of the brain was then washed free of blood by opening the thorax, clamping the descending thoracic aorta, severing both jugular veins, and perfusing 20 ml of lactated Ringer’s solution through the left ventricle of the heart in less than 1 min. After washout, the mouse was immediately decapitated, the brain was removed, and dissected into ten brain regions that were individually weighed. Olfactory bulb was excluded due to variations in collection efficiency. Brains with visible blood after washout were excluded from analysis (*n* = 6). Serum was obtained by centrifuging the carotid artery blood for 10 min at 4000 × *g*. Levels of ^99m^Tc radioactivity in the serum and brain regions were determined in a gamma counter. The same brain regions and sera were then solubilized, and the level of ^14^C radioactivity was determined in a beta counter. Brain tissue radioactivity was calculated by dividing the cpm (gamma), or dpm (beta) in the brain region by the weight of the brain region. Serum radioactivity was calculated by dividing the cpm, or dpm in the serum by the milliliters of serum counted. The brain tissue radioactivity was then divided by the corresponding serum radioactivity and the results given in units of microliters per gram of brain tissue. For shams, no significant differences were observed among the brain/serum ratios at the various points and so were combined and used as a single reference value (*n* = 22).

### Indomethacin and N(G)-nitro-L-arginine methyl ester treatment

In some experiments, mice received three injections of indomethacin (INDO; Sigma; St. Louis MO; 5 mg/kg; 100 µl 7% NaHCO_3_; ip) at 48, 54, and 71 h post-blast. Other mice received three injections of N(G)-nitro-L-arginine methyl ester (L-NAME; Sigma; 10 mg/kg; 100 µl 7% NaHCO_3_; ip) at 48, 54, and 71 h post-blast. For these experiments, Sham controls received identical INDO, or L-NAME injections along with their respective blast cohorts. Vehicle controls received 100 µl injections of 7% NaHCO_3_.

### Confocal microscopy

Dissected mouse brains were immediately post-fixed in 4% paraformaldehyde in PBS at 4 °C and then equilibrated with cryoprotectant (30% sucrose in PBS) overnight at 4 °C. Sagittal sections embedded in OCT (Tissue-Tek, Torrance, CA), and after decay of ^99m^Tc, were cut at 50 µm thickness from bisected brains with a CM1850UV cryostat (Leica, Buffalo Grove, IL). Antigen retrieval was performed with 50 mM sodium citrate (pH 9.0) and then heating at 80 °C for 30 min. Immunostaining was performed according to our previously published methods^14^. Floating tissue sections were cover slipped with a drop of Prolong® Gold Antifade Reagent with or without DAPI (Invitrogen, Eugene, OR). The following antibodies were applied overnight at 4 °C: mouse monoclonal anti-claudin-5 labeled with Alexa 488 (Thermo, Rockford, IL; 1:100), rabbit monoclonal anti-microtubule-associated protein 2 (MAP2; Cell Signaling, Danvers, MA; 1:2000), rabbit polyclonal anti-IBA1 (Wako, Osaka, Japan; 1:1000), chicken polyclonal anti-GFAP (Millipore, Billerica, MA; 1:1000), and rabbit polyclonal CD31 (Abcam, Cambridge, MA; 1:500). Goat secondary antibodies labeled with Alexa 647 and Cy3 were applied for 2 h (Jackson Immunoresearch, West Grove, PA; 1:1000). Confocal microscopy was performed with a Leica TCS SP5 II microscope with Leica objectives (10X and 20X with 0.3 and 0.7 numerical aperture, respectively). Confocal microscopic images were acquired with the Leica Application Suite and processed using image adjustments limited only to linear contrast and brightness adjustments applied identically to data from blast- and sham-treated animals in each experiment.

### Immunoblotting

Left brain hemispheres from mice in the ^99m^Tc-albumin experiments were immediately flash frozen in liquid nitrogen and stored at −80 °C for ten ^99m^Tc-albumin half-lives (~60 h). Hippocampus and striatum were dissected and sonicated in radioimmunoprecipitation assay (RIPA) buffer with protease/phosphatase inhibitors (Thermo; Rockford, IL 1:100). Samples were then centrifuged for 15 min at 12000 × *g* at 4 °C and supernatant collected and standardized with a bicinchoninic acid (BCA) assay. Brain extracts of equivalent total protein content were resolved by sodium dodecyl sulfate-polyacrylamide gel electrophoresis (SDS-PAGE) (20 µg total protein/lane) under reducing conditions, transferred to nitrocellulose, and probed with rabbit polyclonal claudin-5 (Invitrogen, Eugene, OR; 1:1000) and rabbit monoclonal β-actin (Cell signaling, Danvers, MA; 1:10000) as loading controls. Blots were then probed with anti-rabbit horseradish peroxidase-conjugated secondary antibody (Jackson ImmunoResearch; 1:5000) and visualized by enhanced chemiluminescence (GE Healthcare, Piscataway, NJ). Optical densities of the immunoreactive protein bands were quantified using Image-Quant with background subtraction and normalization to loading control.

### Cytokine measurements

Tumor necrosis factor alpha (TNFα) levels were measured using a murine Bio-Plex Pro™ assay kit (Bio-Rad Laboratories, Inc.; Hercules, CA) in brain lysates previously prepared for immunoblotting. All brain samples were diluted 1:3 in sample diluent provided in the kit and processed according to the manufacturer’s protocol. Plates were read on a Bio-Plex 200 (Bio-Rad Laboratories, Inc., Hercules, CA). Values (pg/mg) were acquired by normalizing each sample to their respective BCA concentration obtained for immunoblotting.

### Statistical analysis

Statistical analyses used GraphPad Prism® 6.0 (GraphPad Software, Inc., La Jolla, CA). Error bars represent the standard error of the mean (SEM). Comparison of more than two groups was made using one-way analysis of variance (ANOVA) followed by Dunnett’s *post-hoc* test (for comparing time points to a collective sham group) or Newman-Keuls *post-hoc* test (for comparing experimental groups to sham control groups, and drug treatment groups).

### Data availability

Authors will provide published data and relevant protocols upon request to the Principal Investigator by phone or email.

## Electronic supplementary material

Supplementary Data
