# Supplementary material for: Blast exposure elicits blood-brain barrier disruption and repair mediated by tight junction integrity and nitric oxide dependent processes
Source: Sci Rep. 2018 Jul 27;8:11344. doi: 10.1038/s41598-018-29341-6 (PMC6063850; doi:10.1038/s41598-018-29341-6)
Supplement: Supplementary file 1 — Supplementary Data [file 41598_2018_29341_MOESM1_ESM.docx]

**Blast exposure elicits blood-brain barrier disruption and repair mediated by tight junction integrity and nitric oxide dependent processes**

Aric F. Logsdon, James S. Meabon, Marcella M. Cline, Kristin M. Bullock, Murray A. Raskind, Elaine R. Peskind, William A. Banks & David G. Cook

**Supplementary Figures**

**Supplementary Figure 1. Exposure to primary blast overpressures generated by high explosives was simulated using a well-established pneumatic shock tube.** Red trace denotes the mean waveform of 18 blasts sampled throughout the experiments comprising this report. Blue trace shows an estimated Friedlander waveform expected from detonation of approximately 21kg TNT detonated at a distance of 8m in the open field. Error bars denote SEM.

**
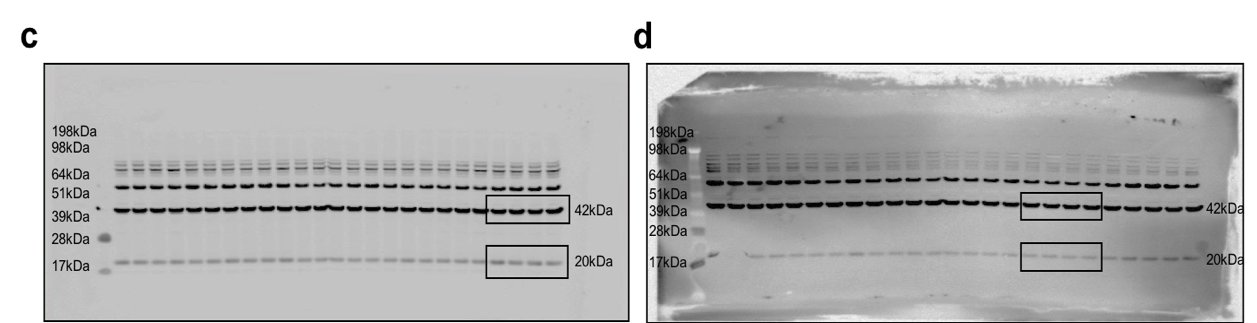
**

**Supplementary Figure 2. No differences were observed in CLD5 protein levels after 2X blast.** Western blot analysis revealed no change in CLD5 protein expression at all time points after blast in **(a)** hippocampus (*p* > 0.05), **(b)** or striatum (*p* > 0.05). One-way ANOVA. Values represent mean ± SEM normalized to β-actin; *n* = 5. **(c)** Inset black boxes demarcate representative CLD5 (20kDa) and β-actin (42kDa) from the uncropped immunoblots in hippocampus, **(d)** and striatum. As previously established under these experimental conditions, the CLD5 antibody does not cross-react with β-actin, nor bind non-specifically at 42kDa, thus both antibodies were immunoblotted together for improved comparisons of total protein per lane.


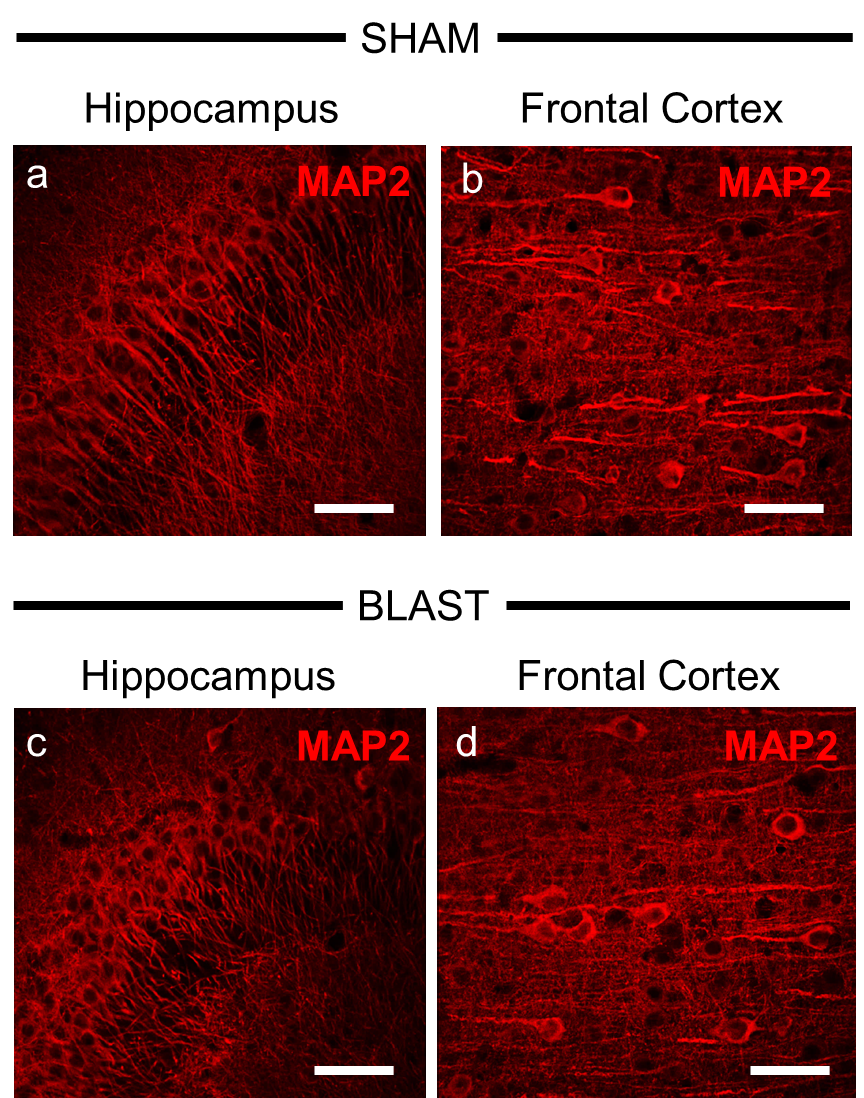


**Supplementary Figure 3. Blast caused no overt changes in hippocampal somato-dendritic neuron morphology at 72 hours after 2X blast.** **(a)** Representative images of MAP2 staining in the hippocampus or **(b)** frontal cortex of sham controls. **(c)** Representative images of MAP2 staining in the hippocampus or **(b)** frontal cortex at 72 h after 2X blast. *n* = 3. Scale bars = 50µm.

**
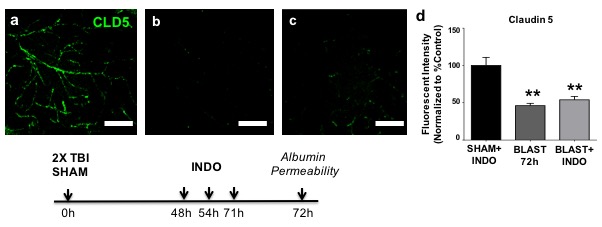
**

**Supplementary Figure 4. Blast-induced loss of claudin-5 immunoreactivity in the CA1 region of the hippocampus was not rescued by indomethacin treatment.** **(a)** Shows representative images of CLD5 staining in hippocampal CA1 region of sham + INDO, **(b)** at 72 h after 2X blast + vehicle, and **(c)** at 72 h after 2X blast + INDO. **(d)** A significant decrease in CLD5 immunofluorescence was measured at 72 h after 2X blast + vehicle (*p* ≤ 0.01), and after 2X blast + INDO administration (*p* ≤ 0.01). One-way ANOVA *post hoc* Newman-Keul’s. Values represent mean±SEM; *n* = 4. (***p* ≤ 0.01 vs sham + INDO). Scale bars = 50µm.
